# What is the postoperative nutrition intake in children with congenital heart disease? A single-center analysis in China

**DOI:** 10.1186/s12887-022-03530-9

**Published:** 2022-08-03

**Authors:** Ping Ni, Mingjie Zhang, Yibei Wu, Wenyi Luo, Zhuoming Xu

**Affiliations:** 1 Department of Cardiothoracic Surgery, Shanghai Children’s Medical Center, School of Medicine, Shanghai Jiao Tong University, Shanghai, China; 2 Nursing Department, Shanghai Children’s Medical Center, School of Medicine, Shanghai Jiao Tong University, Shanghai, China

**Keywords:** Heart Defects, Congenital, Pediatrics, Nutrition, Resting energy expenditure, Caloric intake

## Abstract

**Background:**

It is common that inadequate nutritional intake happens in patients with congenital heart disease (CHD), which can adversely affect the prognosis of patients. However, the details and reasons are not clear enough so far. Therefore, the primary aim of this study was to investigate the current nutritional requirements and energy intake on days 1–7 in the cardiac intensive care unit after surgery. Our secondary aim was to investigate potential factors that hinder nutritional supply and to compare the resting energy expenditure (REE) based on two methods, the Fick method and the Schofield equation.

**Methods:**

Using retrospective analysis, we collected data from postoperative children with CHD at a children's hospital in Shanghai, China. We used the Fick method to calculate the REE, and compare the results with the actual enteral nutrition intake. Meanwhile, we recorded the initiation time of enteral nutrition, feeding intolerance, unfinished milk volume, etc. Then the correlation between the results of the Fick method and the equation method was calculated.

**Results:**

A total of 49 patients were included, with a median age of 22 months (IQR 4.9, 57.3), and a median Aristotle basic complexity score of 8 (IQR 6.0, 9.8). The time interval for surgical intervention within 7 days after operation was 4 (IQR 2.5, 6). No statistical difference in REE on postoperative days 1–7. The average enteral nutrition energy provided 64.6 (33.6, 79.6)% of the REE, which showed a significant decrease on postoperative day 4, and then reached its lowest on postoperative day 5. The protein supply was 0.7 ± 0.3 kcal/kg/d. In addition, the REE calculated by the Fick method was moderately correlated with that estimated by the equation (*r* = 0.467, *P* = 0.001).

**Conclusions:**

The energy and protein supply in the acute postoperative period in children with CHD is inadequate. Fluid restriction and fasting may be the main causes. In addition, there is a moderate correlation between the REE calculated by the Fick method and that estimated by the equation.

**Supplementary Information:**

The online version contains supplementary material available at 10.1186/s12887-022-03530-9.

## Introduction

Congenital heart disease (CHD) is the most common birth defect [1]. In this population, inadequate nutritional intake is a general problem for a variety of reasons [2, 3]. It has been found that early postoperative nutritional support can increase the energy and protein intake of children, improve their nutritional status, so that it can also help to reduce infection, duration of mechanical ventilation and hospital stay [4, 5]. Therefore, adequate nutritional intake in the early postoperative period is crucial for children with CHD. Studies have confirmed that many problems arise in the actual process of nutritional therapy, such as underestimation of energy requirements [6], feeding intolerance [7], delayed initiation of enteral nutrition (EN) or interruption of feeding [8–10], etc., which may affect the nutritional supply of patients after surgery and exacerbate their malnutrition. However, the current status of the above-mentioned problems in children after CHD surgery is unclear.

The European Society of Pediatrics and Neonatal Intensive Care (ESPNIC) recommends use of the Schofield equation to estimate REE in critically ill children in limited conditions [11]. The equation is used to estimate REE from height and weight, and has different reference standards for different ages and genders [12]. On the other hand, the Fick method was proposed in 1987 and it is based on obtaining oxygen consumption (VO_2_) from the patient's thermodilution pulmonary artery catheters, multiplying the VO_2_ by the caloric value for oxygen and thus obtaining the resting energy expenditure (REE) [13].

Therefore, we reviewed data from the past six months, calculated the postoperative REE of patients with the Fick method, and tried to understand the gap between nutritional supply and actual demand in the cardiac intensive care unit (CICU) as well as factors that impede nutritional supply, in order to provide a reference for further optimization of nutritional therapy in children after CHD surgery.

## Methods

### Study design

This is a retrospective cohort study conducted in a CICU with 41 beds in a children's hospital in Shanghai, China. The primary objectives were to understand the current status of postoperative feeding in children with CHD and to compare the gap between actual caloric intake and target requirements. The secondary objectives were to understand the factors that hinder postoperative nutritional availability and to compare the energy prediction equation with the energy requirements calculated by the Fick method. This study was approved by the ethics committee of the Shanghai Children’s Medical Center, and all methods were carried out in accordance with relevant guidelines and regulations.

### Participants

Patients admitted to the CICU from January 2021 to July 2021 were selected. Inclusion criteria were as follows: Children undergoing cardiac surgery; CICU stay of at least three days; and a pulse indicator continuous cardiac output (PiCCO) catheter was used. Patients using total parenteral nutrition (TPN) were excluded.

### Data collection

The general information, disease information and feeding status of the patients were accessed through the hospital information system. General information included gender, age, height and weight, etc. Disease information included diagnosis, surgical procedure, CICU stay, duration of mechanical ventilation, maximum vasoactive inotropic score (VIS) within 48 h postoperatively, cardiac index (CI), and arteriovenous blood gas analysis results, etc. Feeding status included EN type, dose, interruption, and feeding intolerance symptoms such as vomiting and diarrhea. CI was measured by PiCCO device (Pulsion Medical Systems, Feldkirchen, Germany). And blood gas analysis data were from the ABL800 FLEX blood gas analyzer (Radiometer Medical ApS, Denmark).

### Calculation of energy expenditure

According to the Fick method, the REE is calculated using hemoglobin (Hb), cardiac output (CO), arterial oxygen saturation (SaO2), and mixed venous oxygen saturation **(**SvO2**)** with the following formula: the REE (kcal/d) = CO*Hb*(SaO2—SvO2) * 95.18 [13]. The average of the REE at 10:00 AM and 10:00 PM was taken as the patient's REE for the day. Hb, SaO2, and SvO2 were obtained from blood gas analysis data. The REE was then estimated by the Schofield equation. Nutritional intake of at least 80% of REE on any given day in the CICU was defined as reaching the target calories [14]. In addition, blood gas indicators were used to calculate VO_2_ [15].

### Statistical analysis

All data were statistically analyzed using SPSS version 23.0 (IBM, Armonk, NY, USA). Continuous data were tested for normality using the Shapiro–Wilk normality test, expressed as mean ± SD or median (IQR), and categorical data were expressed as rate (%). Exploring the effect of time on REE, protein, and energy using one-way repeated measures ANOVA. Pearson correlation coefficients were also calculated between the REE estimated by the Schofield equation and the measured REE by the Fick method. Statistical significance was set at *P* ≤ 0.05 (two-tailed).

## Results

### Characteristics of the patients

From January 2021 to July 2021, 62 postoperative CHD patients used a PiCCO catheter in the CICU. Five of these patients had a CICU stay of less than three days, and eight patients used TPN during CICU, so these patients were excluded. Finally, forty-nine patients were included. The median age of these children was 22 months and the median length of CICU stay was seven days. Patient information is shown in Table 1. Diagnosis and surgical procedures of the patients are shown in Additional file 1.

**Table 1:** Characteristics of the patients

Characteristics	Results
Male, no. (%)	24 (49.0)
Age (m), Median (IQR)	22.0 (4.9, 57.3)
WAZ score, Median (IQR)	-1.0 (-1.9, 0.0)
HAZ score, Median (IQR)	-0.7 (-1.6, 0.2)
BMIZ score, Median (IQR)	-0.5 (-1.5, 0.1)
ABC score, Median (IQR)	8.0 (6.0, 9.8)
48 h VIS max, Median (IQR)	25.0 (18.8,32.5)
Cardiopulmonary bypass time (min), Median (IQR)	123.5 (82.8, 160.8)
Aortic clamping time (min), Median (IQR)	74.5 (48.0, 100.3)
Mechanical ventilation (h), Median (IQR)	94.5 (50.6, 161.5)
CICU stay (d), Median (IQR)	7.0 (5.0, 9.0)
Hospital stay (d), Median (IQR)	22.0 (17.3, 36.3)
Weight loss during hospitalization (kg), Median (IQR)	0.4 (0.2, 1.0)
Use of blood and its biological products, no. (%)	30 (61.2)
Surgery intervention within 7 days after corrective operation, no. (%)	12 (24.5)
Delayed sternal closure	8 (16.3)
Diaphragm plication	1 (2.0)
Pacemaker placement	1 (2.0)
Mitral valvuloplasty + Pacemaker placement	1 (2.0)
Pericardial drainage	1 (2.0)
Time interval for surgery intervention within 7 days after corrective operation (d), Median (IQR)	4.0 (2.5, 6.0)

*ABC score* Aristotle basic complexity score, *VIS* Vasoactive inotropic score, *WAZ* Weight-for-age z score, *HAZ* Length-for-age z score, *BMIZ* BMI-for-age z score, *IQR* interquartile range

### Characteristics of postoperative energy balance

**Fig. 1:**
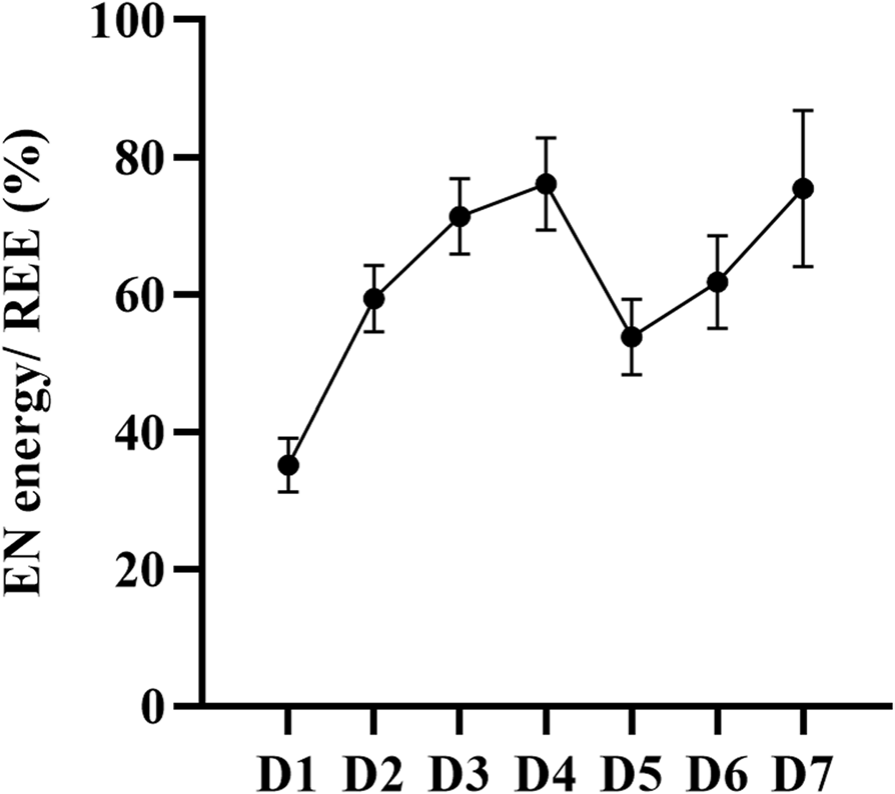
The relationship between enteral nutrition energy supply and resting energy expenditure after surgery

**Fig. 2:**
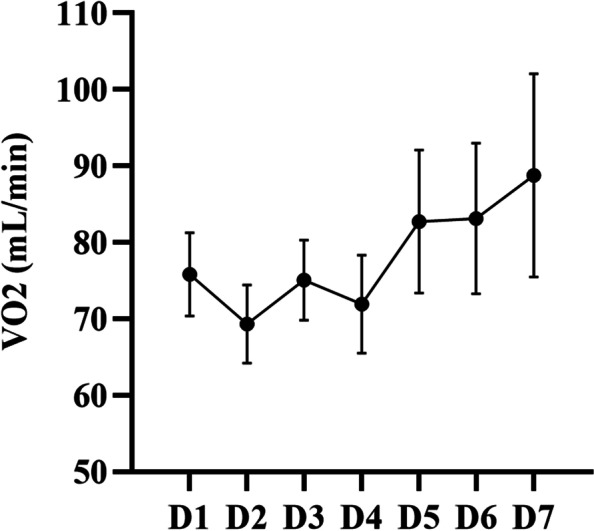
Oxygen consumption after surgery

There was no statistically significant difference between groups for REE 1–7 days postoperatively (*F* = 0.711, *P* = 0.523), but the group comparisons of protein (*F* = 10.625, *P* < 0.001) and energy (*F* = 10.321, *P* < 0.001) on postoperative days 1–7 were statistically different (Table 2). The EN energy supply accounted for 64.6 (33.6, 79.6) % of the REE. Thirty-one patients reached the target calorie, with an attainment rate of 63.3%. The ratio of energy supply to REE showed a tendency to rise, then fall, and then rise again. And the relationship between REE and supplied energy is shown in Fig. 1. Besides, Fig. 2 demonstrates the change in VO_2_ after cardiac surgery, and the trend is consistent with that of the REE. Thirty children were transfused with blood and its biologics on days 1–7 after surgery. The trend of blood product use was the opposite, showing a trend of first decreasing, then increasing on days 4 to 5, and finally decreasing again (Additional file 2).

**Table 2:** Changes in resting energy expenditure, protein and energy on postoperative days 1–7

	D1	D2	D3	D4	D5	D6	D7	*F*	*P*
REE(kcal/kg/d), mean ± SD	45.4 ± 20.3	40.3 ± 13.2	46.3 ± 18.0	41.4 ± 13.5	48.7 ± 15.7	44.4 ± 18.6	49.3 ± 13.1	0.711	0.523
Protein(g/kg/d), mean ± SD	0.37 ± 0.27	0.67 ± 0.47	0.81 ± 0.44	0.79 ± 0.41	0.67 ± 0.47	0.84 ± 0.41	1.01 ± 0.39	10.625	< 0.001
Energy(kcal/kg/d), mean ± SD	13.6 ± 10.2	23.4 ± 13.5	32.2 ± 17.1	30.9 ± 15.9	26.7 ± 17.1	31.8 ± 15.0	37.1 ± 14.6	10.321	< 0.001

*REE* Resting energy expenditure, *SD* Standard deviation

### Information related to enteral nutrition

In terms of symptoms of feeding intolerance, no patient experienced vomiting, and the percentage of diarrhea and fecal occult blood was 2.0% and 6.1%, respectively. Patients were in negative fluid balance with an average of -19.8 ± 8.2 mL/kg/d. For EN, the average unscheduled milk stoppage was 12.2 ± 7.7 mL/kg/d, with a protein supply of 0.7 ± 0.3 g/kg/d, much lower than 1.5 g/kg/d (Table 3).

**Table 3:** Characteristics of postoperative enteral nutrition

Characteristics	Results
Vomiting, no. (%)	0 (0)
Diarrhea, no. (%)	1 (2.0)
Fecal occult blood, no. (%)	3 (6.1)
Use of high energy formulas^a^, no. (%)	37 (75.5)
Feeding methods, no. (%)	
Gastric tube	19 (38.8)
Gastric tube + oral feeding	30 (61.2)
Accumulated fluid balance (mL/kg/d), mean ± SD	-19.8 ± 8.2
Time of EN initiation (h), mean ± SD	26.2 ± 7.2
Unfinished milk volume (mL/kg/d), mean ± SD	12.2 ± 7.7
EN protein intake (g/kg/d), mean ± SD	0.7 ± 0.3

*EN* Enteral nutrition, *SD* Standard deviation

^a^refers to formula with an energy density of 100 kcal/100 mL

In addition, we calculated the correlation between the REEs obtained by the two methods. The correlation between the REE estimated using the Schofield equation and that calculated using the Fick method was moderate (*r* = 0.467, *P* = 0.001).

## Discussion

ESPNIC recommends the use of the indirect calorimetry (IC) method to measure REE for guiding nutritional support in critically ill infants [11]. The IC method is based on the oxygen and carbon dioxide in the inhaled and exhaled gases per unit of time, calculates the VO_2_ and carbon dioxide production (VCO_2_), and then calculates the energy expenditure by Weir's formula [16]. However, the IC method is not widely used in the CICU due to its high cost, the need for specialized training of the operator, and some high requirements in practice [17]. The Fick method correlates well with the IC method (*r* = 0.9, *P* < 0.0001) and can be used to measure the REE in critically ill patients [13]. Because of the invasive operation, this study used a retrospective analysis to include patients who used a PiCCO catheter, a common alternative to pulmonary artery catheters placed in pediatric patients [18].

In a study by Zhang et al. [19], the REE of patients with a mean age of 3 months measured by indirect calorimeter was 55–57 kcal/kg/d on days 1–4 after cardiopulmonary bypass. Li et al. [20] used modified Weir’s equation to calculate the REE on days 1–3 after the Norwood procedure, and the REE was 39–41 kcal/kg/d in patients aged 4–92 days. In our study, the mean REEs on postoperative days 1–7 was 40–49 kcal/kg/d. But the patients in our study were 22 months old and had an Aristotle basic complexity (ABC) score of 8, which was different from the above study population and less comparable. We found that children had a higher REE on the first postoperative day, which was associated with an increased stress response in the early postoperative period. In addition, the REE was also higher after the fifth postoperative day, which, in addition to the reason for the stress response from the re-intervention, was also explained by the decrease in the use of sedative drugs as the tracheal tube was removed. However, there was no statistical difference in REE on different days postoperatively.

Our study found that energy supplied through EN did not account for a high proportion of patients' REE. Nicholson et al. [21] also found that the actual caloric intake of neonates who underwent modified systemic-to pulmonary artery shunt was much lower than the recommended intake. And the ratio of EN energy supply to REE started to decrease on day four and reached its lowest on day five. In addition to inadequate energy, protein supply in postoperative CHD patients is even more inadequate. For critically ill children requiring EN, the daily enteral protein intake is at least 1.5 g/kg [11, 22]. Even one study found that the protein requirement of children with CHD in the early post-cardiopulmonary bypass period is about 4 g/kg/d. High protein feeding can reverse the negative nitrogen balance earlier and improve nutritional outcomes [19]. However, in our study, the highest protein intake on postoperative days 1–7 was 1.01 g/kg/d, with an average of 0.7 g/kg, which was much lower than the actual requirement.

There are several possible reasons for such a low nutritional supply in children with CHD. The first is fluid restrictions. Children with CHD have strict fluid restriction after surgery to prevent cardiac overload [23]. In addition, while conservative feeding practices are beneficial in reducing intolerance, they may, on the other hand, further reduce nutritional intake. Therefore, faced with the conflict between fluid restriction and insufficient intake, high energy formula can provide patients with more nutrition [24, 25]. It is also possible to take a cue from another study and add pure whey protein isolate to formula [19].

The second point is fasting before and after surgical intervention or extubation. It should be noted that in our CICU, it is usually those patients who require close hemodynamic monitoring who are on PiCCO. This study retrospectively included children who had been on PiCCO, so these individuals tended to be sicker and had higher rates of re-intervention. The median time of re-intervention was 4 days, a critical time point, which on the one hand would be preceded by fasting, and on the other hand, would be followed by a higher infusion of blood products, and a reduction in EN intake was often necessary to ensure that total fluid intake was not excessive. In addition, the median duration of mechanical ventilation is almost 4 days, and fasting also occurs before and after removal of the tracheal tube. Despite guideline recommendations, there is still a large gap between theory and practice, which requires evidence-based clinical improvements to facilitate the translation of recommended preoperative fasting guidelines into clinical practice [26].

The third point is the initiation time of EN. The initiation time of EN was slightly longer than 24 h. This time, of course, includes the time from postoperative admission to the CICU until the patient receives the EN, the time of preparation and delivery by the nutrition department, and the time of delay in distributing the EN within the CICU. So, for the delay between the time the EN is ordered and the time the EN is started, we may be able to do some quality improvement projects to facilitate the early start of the EN.

Furthermore, the amount of unfinished milk was 12.2 ± 7.7 mL/kg/d. Lee et al. [9] classified the causes of nutritional interruptions into four categories, i.e., procedures, intolerance, potentially avoidable, and unknown. As this study was retrospective, the specific causes of these nutritional interruptions were not recorded. Thus, future prospective investigations are recommended to comprehensively investigate the factors influencing the nutritional supply deficiencies to provide a reference for intervention.

The Schofield equation is a method recommended by ESPNIC to estimate REE, but it only relates to height, weight, sex, and age, which cannot dynamically estimate the REE of critically ill patients. Some studies have suggested that the equation estimates of REE are highly biased [6, 27]. We found a moderate correlation coefficient of 0.467 between the REE calculated by the Fick method and the REE estimated by the equation. Therefore, the accuracy of the equation in children with CHD needs to be further verified.

There are some limitations to this study. The interpretation of the study findings is somewhat limited by the fact that it is a single-center, small-sample study. In addition, the retrospective design allows for limited data collection, which affects the interpretation of the influencing factors. Therefore, a more comprehensive exploration of the factors affecting nutrition supply is needed in the future.

## Conclusions

In our study, the energy and protein supply in the early postoperative period in children with CHD was inadequate, especially the protein supply. Strict fluid restriction, as well as fasting, are possible causes. In addition, we found a moderate correlation between the REE calculated by the Fick method and the REE estimated by the Schofield equation.

## Supplementary Information

**Additional file 1.** Patient diagnosis and surgical procedure.

**Additional file 2.** Use of blood and its biological products after surgery.

## Data Availability

Data can be made available on request and following institutional and ethic board approvals for release.

## Abbreviations

CHD: Congenital heart disease
REE: Resting energy expenditure
EN: Enteral nutrition
CICU: Cardiac intensive care unit
PiCCO: Pulse indicator continuous cardiac output
TPN: Total parenteral nutrition
VIS: Vasoactive inotropic score
CI: Cardiac index
Hb: Hemoglobin
CO: Cardiac output
SaO2: Arterial oxygen saturation
SvO2: Mixed venous oxygen saturation
VO_2_: Oxygen consumption
ABC score: Aristotle basic complexity score
WAZ: Weight-for-age z score
HAZ: Length-for-age z score
BMIZ: BMI-for-age z score
IQR: Interquartile range
SD: Standard deviation
IC: Indirect calorimetry
VCO_2_: Carbon dioxide production

## References

[1] Pierpont ME, Brueckner M, Chung WK (2018). Genetic basis for congenital heart disease: revisited: a scientific statement from the American heart association. Circulation.

[2] Vieira T, Trigo M, Alonso R (2007). Assessment of food intake in infants between 0 and 24 months with congenital heart disease. Arq Bras Cardiol.

[3] Hong BJ, Moffett B, Payne W (2014). Impact of postoperative nutrition on weight gain in infants with hypoplastic left heart syndrome. J Thorac Cardiovasc Surg.

[4] Sahu MK, Singal A, Menon R (2016). Early enteral nutrition therapy in congenital cardiac repair postoperatively: a randomized, controlled pilot study. Ann Card Anaesth.

[5] Du N, Cui Y, Xie W (2021). Application effect of initiation of enteral nutrition at different time periods after surgery in neonates with complex congenital heart disease: a retrospective analysis. Medicine.

[6] Vest MT, Newell E, Shapero M (2019). Energy balance in obese, mechanically ventilated intensive care unit patients. Nutrition.

[7] Tume LN, Valla FV (2018). A review of feeding intolerance in critically ill children. Eur J Pediatr.

[8] Solana MJ, Manrique G, Fernández R (2020). Nutritional status and nutrition support in critically ill children in Spain: Results of a multicentric study. Nutrition.

[9] Lee ZY, Ibrahim NA, Mohd-Yusof BN (2018). Prevalence and duration of reasons for enteral nutrition feeding interruption in a tertiary intensive care unit. Nutrition.

[10] Leong AY, Cartwright KR, Guerra GG, Joffe AR, Mazurak VC, Larsen BMK (2014). A Canadian survey of perceived barriers to initiation and continuation of enteral feeding in PICUs. Pediatr Crit Care Med.

[11] Tume LN, Valla FV, Joosten K (2020). Nutritional support for children during critical illness: European Society of Pediatric and Neonatal Intensive Care (ESPNIC) metabolism, endocrine and nutrition section position statement and clinical recommendations. Intensive Care Med.

[12] Schofield WN (1985). Predicting basal metabolic, new standards and review of previous work. Hum Nutr Clin Nutr.

[13] Liggett SB, St John RE, Lefrak SS (1987). Determination of resting energy expenditure utilizing the thermodilution pulmonary artery catheter. Chest.

[14] Ni P, Chen X, Zhang Y (2022). High-energy enteral nutrition in infants after complex congenital heart surgery. Front Pediatr.

[15] Scheeren TWL, Wicke JN, Teboul JL (2018). Understanding the carbon dioxide gaps. Curr Opin Crit Care.

[16] Weir JB (1949). New methods for calculating metabolic rate with special reference to protein metabolism. J Physiol.

[17] Zhang J, Cui YQ, Ma Md ZM, Luo Y, Chen XX, Li J (2019). Energy and protein requirements in children undergoing cardiopulmonary bypass surgery: current problems and future direction. JPEN J Parenter Enteral Nutr.

[18] De Backer D, Bakker J, Cecconi M (2018). Alternatives to the Swan-Ganz catheter. Intensive Care Med.

[19] Zhang J, Cui YQ, Luo Y, Chen XX, Li J (2021). Assessment of energy and protein requirements in relation to nitrogen kinetics, nutrition, and clinical outcomes in infants receiving early enteral nutrition following cardiopulmonary bypass. JPEN J Parenter Enteral Nutr.

[20] Li J, Zhang GC, Herridge J (2008). Energy expenditure and caloric and protein intake in infants following the Norwood procedure. Pediatr Crit Care Med.

[21] Nicholson GT, Clabby ML, Kanter KR, Mahle WT (2013). Caloric intake during the perioperative period and growth failure in infants with congenital heart disease. Pediatr Cardiol.

[22] Mehta NM, Skillman HE, Irving SY (2017). Guidelines for the provision and assessment of nutrition support therapy in the pediatric critically ill patient: society of critical care medicine and american society for parenteral and enteral nutrition. JPEN J Parenter Enteral Nutr.

[23] Hanot J, Dingankar AR, Sivarajan VB (2019). Fluid management practices after surgery for congenital heart disease: a worldwide survey. Pediatr Crit Care Med.

[24] Chen X, Zhang MJ, Song YX (2021). Early high-energy feeding in infants following cardiac surgery: a randomized controlled trial. Transl Pediatr.

[25] Kalra R, Vohra R, Negi M (2018). Feasibility of initiating early enteral nutrition after congenital heart surgery in neonates and infants. Clin Nutr ESPEN.

[26] Li Y, Lu Q, Wang B (2021). Preoperative fasting times for patients undergoing elective surgery at a pediatric hospital in shanghai: the big evidence-practice gap. J Perianesth Nurs.

[27] De Cosmi V, Mehta NM, Boccazzi A (2017). Nutritional status, metabolic state and nutrient intake in children with bronchiolitis. Int J Food Sci Nutr.

