# Supplementary material for: What is the postoperative nutrition intake in children with congenital heart disease? A single-center analysis in China
Source: BMC Pediatr. 2022 Aug 3;22:470. doi: 10.1186/s12887-022-03530-9 (PMC9347112; doi:10.1186/s12887-022-03530-9)
Supplement: Supplementary file 1 — Additional file 1. Patient diagnosis and surgical procedure. [file 12887_2022_3530_MOESM1_ESM.docx]

**Additional file 1.** Patient diagnosis and surgical procedure

| **Diagnosis, n (%)** | **Surgical procedures, n (%)** | | | |
| --- | --- | --- | --- | --- |
| Tetralogy of Fallot, 10 (20.4) | Tetralogy repair, 10 (20.4) | | | |
| Coronary artery origin anomalous, 8 (16.3) | ALC-PA repair, 4 (8.2) | Left coronary artery grafting with pedicle, 2 (4.1) | Coronary artery repair, 1 (2) | Coronary artery bypass, 1 (2) |
| VSD, 6 (12.2) | VSD membranous repair, 6 (12.2) | | | |
| Mitral insufficiency, 4 (8.2) | Mitral valvuloplasty, 3 (6.1) | Mitral replacement, 1 (2) | | |
| DORV, 4 (8.2) | DORV repair, 2 (4.1) | Conduit RV-PA, 1 (2) | Arterial switch repair, 1 (2) | |
| Aortic insufficiency, 3 (6.1) | Ross procedure, 2 (4.1) | Bentall procedure, 1 (2) | | |
| LVOT stenosis, 3 (6.1) | Morrow operation, 2 (4.1) | Subaortic myectomy, 1 (2) | | |
| D-TGA with VSD, 2 (4.1) | Rastelli operation, 2 (4.1) | | | |
| Pulmonary atresia with VSD, 2 (4.1) | Pulmonary atresia/VSD repair, 1 (2) | PA unifocalization, 1 (2) | | |
| Coarctation, 2 (4.1) | Aortic arch repair, 2 (4.1) | | | |
| Pulmonary valve absent, 1 (2) | Pulmonary valvuloplasty, 1 (2) | | | |
| Residual stenosis, 1 (2) | Right ventricular outflow tract reconstruction, 1 (2) | | | |
| Aortic stenosis, 1 (2) | Aortic valvotomy, 1 (2) | | | |
| Supravalvar aortic stenosis, 1 (2) | Aortic arch repair, 1 (2) | | | |
| Pulmonary dysplasia, 1 (2) | Pulmonary arterioplasty, 1 (2) | | | |

VSD: ventricular septal defect; DORV: double outlet right ventricle; LOVT: left ventricular outflow tract; TGA: transposition of the great arteries; PA: pulmonary artery; RV: right ventricle.
