# Supplementary material for: What is the postoperative nutrition intake in children with congenital heart disease? A single-center analysis in China
Source: BMC Pediatr. 2022 Aug 3;22:470. doi: 10.1186/s12887-022-03530-9 (PMC9347112; doi:10.1186/s12887-022-03530-9)
Supplement: Supplementary file 2 — Additional file 2. Use of blood and its biological products after surgery. [file 12887_2022_3530_MOESM2_ESM.pdf]

Additional file 2. Use of blood and its biological products after surgery

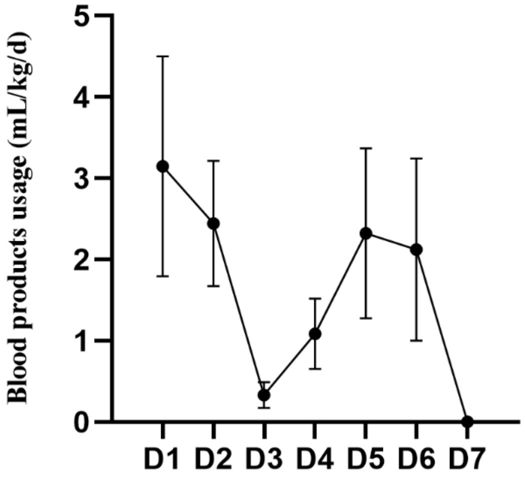

The trend of blood product use showed a trend of first decreasing, then increasing on days 4 to 5, and finally decreasing again
